# Effects of Normobaric Hypoxia of Varying Severity on Metabolic and Hormonal Responses Following Resistance Exercise in Men and Women

**DOI:** 10.3390/jcm14051514

**Published:** 2025-02-24

**Authors:** Jakub Foltyn, Kamila Płoszczyca, Miłosz Czuba, Adam Niemaszyk, Józef Langfort, Robert Gajda

**Affiliations:** 1 Department of Sports Theory, Jerzy Kukuczka Academy of Physical Education, 40-065 Katowice, Polandj.langfort@awf.katowice.pl (J.L.); 2 Faculty of Rehabilitation, Józef Piłsudski University of Physical Education in Warsaw, 00-968 Warsaw, Poland; kamila.ploszczyca@awf.edu.pl (K.P.); adam.niemaszyk@awf.edu.pl (A.N.); 3 Department of Applied and Clinical Physiology, Collegium Medicum, University of Zielona Gora, 65-046 Zielona Góra, Poland; 4 Department of Kinesiology and Health Prevention, Jan Dlugosz University, 42-200 Czestochowa, Poland; 5 Center for Sports Cardiology at the Gajda-Med Medical Center in Pultusk, 06-100 Pultusk, Poland

**Keywords:** hypoxia, resistance exercise, hormonal response, anabolic hormones

## Abstract

**Background/Objectives**: Resistance exercise under hypoxic conditions induces various metabolic and hormonal responses, yet the relationship between hypoxia severity and anabolic hormone responses remains unclear. This study aimed to assess the effects of a single bout of resistance exercise on metabolic and hormonal responses in normoxia and three levels of hypoxia in both men and women. **Methods**: The study involved 16 physically active individuals with at least two years of experience in recreational resistance training. The participants completed resistance exercise sessions in normoxia and normobaric hypoxia at simulated altitudes of 3000 m (H3000), 4000 m (H4000), and 5000 m (H5000). Blood levels of total testosterone (T), cortisol (C), growth hormone (GH), and metabolic variables were measured before and after exercise. **Results**: In women, severe hypoxia (H4000 and H5000) was found to significantly enhance post-exercise increases in T and GH compared to H3000 (*p* < 0.05), without affecting C levels. In men, hypoxia (regardless of intensity) did not significantly augment post-exercise changes in T and GH compared to normoxia. In H4000 conditions, an increase in C levels was observed (*p* < 0.05), leading to an unfavorable reduction in the T/C ratio. Additionally, a reduction in the total number of repetitions performed during the training session and a weakened metabolic response (lactate and creatine kinase) were observed in men at H5000. **Conclusions**: In women, severe hypoxia (H5000) was found to induce a pronounced hormonal response, particularly in GH levels. The use of severe hypoxia during resistance exercise appears unfavorable in men due to a reduced metabolic response, and diminished exercise capacity, coupled with a failure to induce more favorable changes in the secretion of anabolic hormones than in normoxic conditions.

## 1. Introduction

Resistance exercise elicits both acute and chronic hormonal responses that are crucial for muscular adaptations. The heightened secretion of growth hormone (GH) and testosterone (T) in response to resistance training supports muscle growth and remodeling, leading to increased muscle mass and strength [1]. Resistance exercise combined with blood flow restriction (BFR) has been found to be an effective training method for promoting muscle strength and hypertrophy [2,3], and the local hypoxic and ischemic environment is considered to be an important factor in boosting both acute and chronic responses to resistance exercise, including the secretion of anabolic hormones through the local accumulation of metabolites [2,4]. These findings prompted further investigation into how systemic hypoxia affects metabolic and hormonal responses to resistance training. Manimmanakorn et al. [5] demonstrated that both blood flow restriction (BFR) training and training in a systemic hypoxic environment resulted in substantial improvements in muscular strength compared to traditional resistance training performed in normoxia.

It appears that to effectively induce muscle adaptations through resistance training, a high level of metabolic stress must be created during each exercise session [6,7]. It has been shown that the accumulation of metabolic byproducts leads to increased muscle cell swelling, hypertrophic signaling, and motor unit recruitment [8,9,10]. Moreover, higher metabolic stress influences increased post-exercise concentrations of anabolic hormones [8].

Increased metabolic and hormonal responses to resistance exercise can be achieved through various combinations of exercise intensity, number of sets and repetitions, and rest period duration [11], as well as through an additional stress factor such as hypoxia [12]. However, for the effective design of resistance training programs in hypoxia, it is first necessary to consider whether additional oxygen deprivation will actually have a positive impact on metabolic and hormonal responses to exercise.

Previous studies on acute hormonal and metabolic responses to resistance exercise under hypoxia have produced mixed results, showing both benefits and no benefits compared to training in normoxia [13,14,15,16,17,18]. Additionally, the studies vary in terms of hypoxia levels and exercise protocols, making it difficult to interpret the data. In addition, the studies conducted so far have involved almost exclusively men, leaving the potential differences in how women respond to resistance training [19,20] and hypoxia [21] unexplored.

It remains unclear if the severity of hypoxia is associated with the strength of anabolic hormonal responses to resistance exercise. Recently, Jiang et al. [22] observed that only severe hypoxia (SpO_2_ = 80%), but not moderate hypoxia (SpO_2_ = 90%), during high-load resistance exercise increases metabolic and hormonal responses (especially GH) compared to normoxia. Previous studies have shown that resistance exercise (50–85% 1RM) under conditions of severe hypoxia (FiO_2_ = 13.0%) led to increased blood lactate accumulation as well as a more pronounced GH response than exercise under normoxia [13,14]. However, Ramos-Campo et al. [23] noted that severe hypoxia (FiO_2_ = 13%) combined with high-intensity resistance training may cause greater fatigue, leading to a reduction in muscle strength and exercise capacity, ultimately negatively affecting the training stimulus during the session.

To the best of our knowledge, no previous studies have analyzed the effects of combining three levels of systemic hypoxia (ranging from moderate to very severe) with resistance exercise. Therefore, the aim of our study was to analyze metabolic and hormonal variables in response to normoxia and hypoxia of varying severity (FiO_2_ = 14.4%, 12.7%, 11.2%) during high-intensity resistance exercise in physically active men and women with experience in recreational resistance training. Additionally, we examined whether combining high-intensity resistance exercise with severe hypoxia might negatively impact exercise capacity and participants’ ability to properly perform exercises during the training session.

## 2. Materials and Methods

### 2.1. Participants

The sample size was determined through a statistical power analysis (G*Power 3.1). The analysis revealed that a sample of *n* = 8 with an acceptable power of (1 − β = 0.80) and α = 0.05 would be sufficient to detect an effect size greater than 0.50.

The study included 8 men (age, 24.1 ± 0.6 years; body height, 177.0 ± 4.4 cm; body mass, 79.4 ± 9.7 kg; body fat percentage, %FAT 11.9 ± 2.6%; lean body mass, FFM 69.9 ± 7.6 kg) and 8 women (age 24.5 ± 0.9 years; body height, 164.3 ± 2.2 cm; body mass, 62.8 ± 8.8 kg; body fat percentage, %FAT 26.2 ± 4.2%; lean body mass, FFM 46.0 ± 3.8 kg).

The participants had engaged in recreational resistance training for at least 2 years prior to the study and had not been exposed to hypoxia in the 6 months leading up to the study. All participants declared that they had not used any agents included in the World Anti-Doping Agency’s (WADA) prohibited list. Female participants reported regular menstruation and declared that they were not using hormonal contraceptives. Additionally, all participants had current medical examinations, which confirmed their good health and capacity to undertake intensive physical exercise.

Before the commencement of the study, all participants were informed of the purpose and course of the study and provided their written informed consent to participate. Participants were also advised of their right to withdraw from the experiment at any stage without providing any reasons. The research project was conducted according to the Helsinki Declaration and was approved (no. 10/2015) by the Ethics Committee for Scientific Research at the Jerzy Kukuczka Academy of Physical Education in Katowice, Poland.

### 2.2. Study Design

The study employed a blinded crossover design and spanned a total of 4 weeks, during which participants made 5 visits to the laboratory. During the first visit, each participant’s one-repetition maximum (1RM) was determined under normoxic conditions. After 3 days, participants completed their first resistance exercise session. The conditions in which they performed resistance exercises were assigned randomly. Over the course of 4 weeks, the participants participated in 4 sessions, occurring 7 days apart in the conditions of normoxia and normobaric hypoxia at simulated altitudes of 3000 m (FiO_2_ = 14.4%; H3000), 4000 m (FiO_2_ = 12.7%; H4000), and 5000 m (FiO_2_ = 11.2%; H5000). Normobaric hypoxic conditions were generated using the LOS-HYP-1/3NU climate system (Lowoxygen Systems, Berlin, Germany). The sessions began at 9 a.m., with the participants entering the trials in a predetermined order to avoid the impact of diurnal variations in hormone concentrations.

### 2.3. 1RM Test

To determine 1RM for the barbell squat, the participants began with warm-up sets at approximately 50% and 70% of their estimated RM for 5–10 repetitions. Following the warm-up, they performed 4–5 trials with progressively heavier weights, allowing for a 3 min rest interval between the trials. The goal was to complete 3–5 repetitions with the maximum weight. Participants were instructed to perform each exercise at a comfortable pace, and repetition was considered valid if the squat was performed with 90-degree knee flexion. A 3 min rest interval was observed between trials. The 1RM weight was calculated using a formula developed by Brzycki [24].

### 2.4. Study Series

The first study series began with fasting measurements of the participants’ body mass and composition using a bioelectrical impedance analysis (InBody 570, Biospace, Seoul, Republic of Korea). The participants then ate a controlled mixed meal breakfast 3 h before the study. Blood samples were collected at three points: after 15 min of rest in the chamber (rest), immediately following exercise (max), and 60 min after completing exercise (1 h after). Total testosterone (T), cortisol (C), and growth hormone (GH) were determined from venous blood, while creatine kinase (CK), lactate dehydrogenase (LDH), uric acid (UA), and lactate (LA) were measured from capillary blood. The resistance exercise protocol included 10 sets of 12 repetitions at an intensity of 70% of 1RM for the barbell squat, with 3 min rest periods between the sets. The protocol was preceded by a 10 min warm-up. If participants were unable to complete the required number of repetitions, they paused and then resumed the set after a recovery break to complete the 10 sets. The period from the end of the final set to the last blood draw (60 min) was spent in the laboratory under the same conditions as during exercise.

### 2.5. Blood Collection and Analysis

Venous blood (10 mL) was drawn from a basilic vein, and capillary blood samples were collected for LA measurement. Levels of T and C in blood were measured using Roche’s electrochemiluminescence immunoassay on the Cobas analyzer. GH concentrations were determined via immunoradiometric assay using a DSL-1900 IRMA kit (Diagnostic System Laboratories, Webster, TX, USA). LA levels in blood were assessed using a Biosen C-line Clinic analyzer (EKF-diagnostic GmbH, Barleben, Germany). Measurements of CK, LDH, and UA were performed using a Piccolo Xpress biochemical analyzer (Abaxis, Parsipanny, NJ, USA). To account for changes in plasma volume, hematocrit values were also measured. Changes in plasma volume (ΔPV%) were factored into the post-exercise analysis of all biochemical variables and calculated using the formula proposed by van Beaumont [25].

### 2.6. Statistical Methods

The normality of the data distributions was assessed with the Shapiro–Wilk test. Homogeneity of variance was tested using Levene’s test. The analysis was performed separately for the male and the female group. To determine the significance of differences between consecutive measurement points (rest, max, and 1 h after) and between conditions (normoxia and H3000, H4000, and H5000), repeated measures analysis of variance (ANOVA) was used. Significant differences identified by ANOVA were analyzed using the Tukey’s post hoc test. For non-normally distributed variables, the hypotheses were tested using non-parametric tests: Friedman’s ANOVA with a post hoc test. A significance level of *p* < 0.05 was set for all tests. Data are presented as means with standard deviations, and medians (for GH). All computations were performed using Statistica v.13 (TIBCO Software Inc., Palo Alto, CA, USA).

## 3. Results

### 3.1. Load During Study Trials

**Figure 1:**
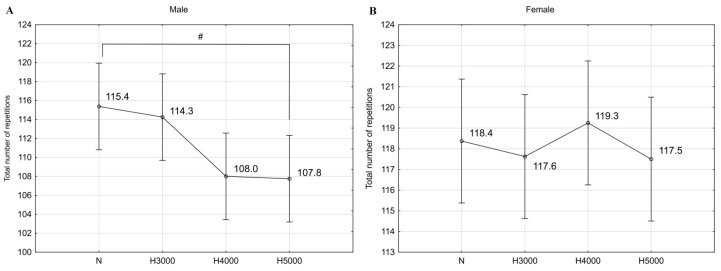
Total number of repetitions completed during a training unit in normoxia and various levels of hypoxia in the male group (**A**) and in the female group (**B**). # *p* < 0.09.

For training units performed in normoxia and hypoxia, the mean absolute load was 88.1 ± 14.4 kg (1.11 ± 0.11 kg/kg BM) for the male group and 50.6 ± 10.1 kg (0.82 ± 0.18 kg/kg BM) for the female group. In men, there was a statistically significant effect of simulated altitude on the total number of repetitions performed during the training session (F = 3.28; *p* < 0.05). In this group, there was a trend toward a decrease (*p* < 0.09) in the total number of repetitions under H5000 conditions compared to normoxia. In the female group, altitude did not affect the total number of repetitions performed (Figure 1).

### 3.2. Testosterone

**Figure 2:**
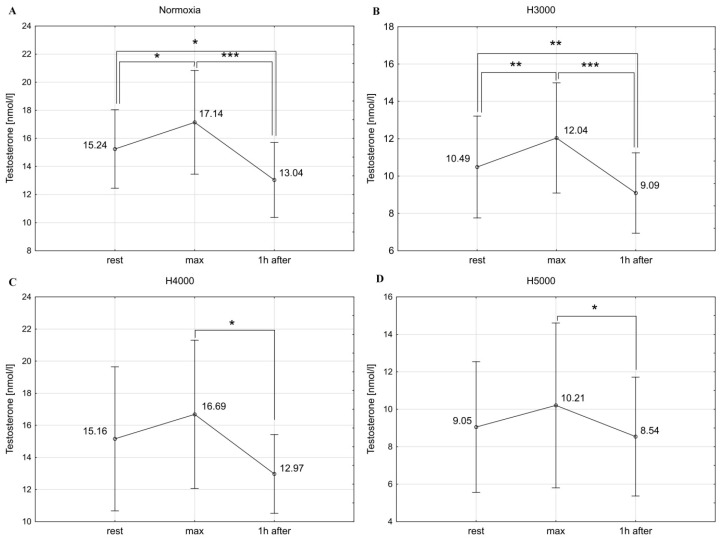
Blood T levels before (rest), immediately after (max), and 1 h after resistance exercise in normoxia (**A**) and hypoxia at simulated altitudes of 3000 m (**B**), 4000 m (**C**), and 5000 m (**D**) in the male group. * *p* < 0.05; ** *p* < 0.01; *** *p* < 0.001.

In the male group, resistance exercise caused significant changes in T concentrations in normoxia (F = 21.08; *p* < 0.001) and at simulated altitudes of H3000 (F = 27.07; *p* < 0.001), H4000 (F = 5.04; *p* < 0.05), and H5000 (F = 4.85; *p* < 0.05). Under normoxia and H3000, there was a significant increase in T immediately after exercise, by 12.5% (*p* < 0.05) and 14.8% (*p* < 0.01), respectively. One hour after the end of exercise, T levels significantly decreased, reaching values lower than baseline (Figure 2). Under the conditions of H4000 and H5000, T concentrations immediately after exercise remained unchanged, while 1 h after exercise T decreased significantly (*p* < 0.05) compared to maximum values (Figure 2).

**Figure 3:**
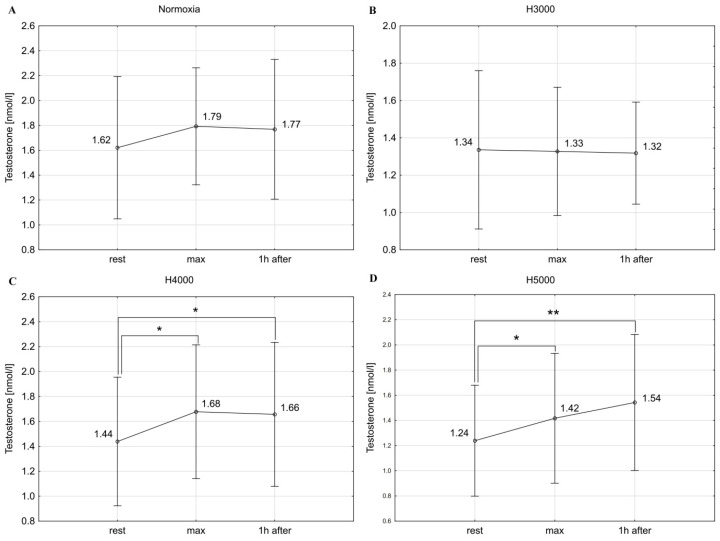
Blood T levels before (rest), immediately after (max), and 1 h after resistance exercise in normoxia (**A**) and hypoxia at simulated altitudes of 3000 m (**B**), 4000 m (**C**), and 5000 m (**D**) in the female group. * *p* < 0.05; ** *p* < 0.01.

The female group demonstrated a significant effect of resistance exercise on T concentrations under H4000 (F = 5.15; *p* < 0.05) and H5000 (F = 12.43; *p* < 0.01). Specifically, T concentrations increased significantly (*p* < 0.05) immediately after exercise by 16.7% (H4000) and by 14.5% (H5000) and remained significantly higher than resting values 1 h after exercise. Under normoxia and H3000, no statistically significant changes in T concentrations in response to resistance exercise were observed (Figure 3).

In the male group, the level of hypoxia did not affect the magnitude of change in T concentration (delta T) in response to resistance exercise. In the female group, the increase in T immediately after exercise was significantly greater at H4000 (*p* < 0.01) and H5000 (*p* < 0.05) than at H3000 (Table 1).

### 3.3. Cortisol

**Figure 4:**
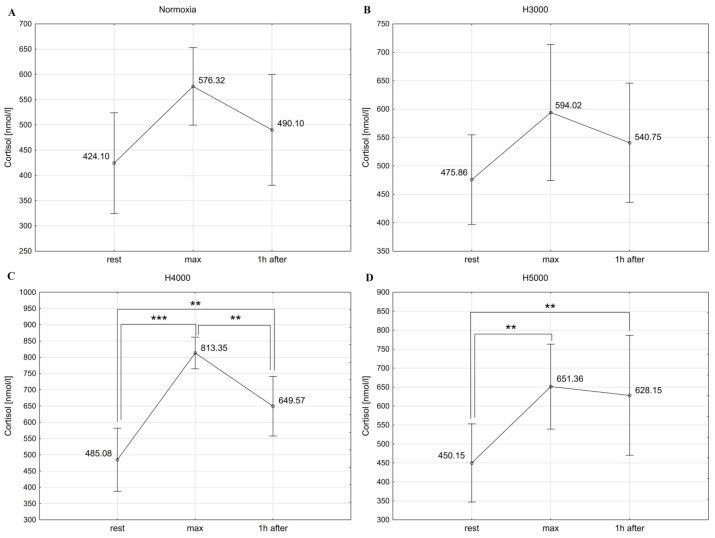
Blood C levels before (rest), immediately after (max), and 1 h after resistance exercise in normoxia (**A**) and hypoxia at simulated altitudes of 3000 m (**B**), 4000 m (**C**), and 5000 m (**D**) in the male group. ** *p* < 0.01; *** *p* < 0.001.

In the male group, resistance exercise led to significant changes in C concentrations at simulated altitudes of H4000 (F = 29.50; *p* < 0.001) and H5000 (F = 13.04; *p* < 0.001). Immediately after exercise, C increased by 67.7% (*p* < 0.001) under H4000 and by 44.7% (*p* < 0.01) under H5000. Under H4000, C dropped (*p* < 0.01) 1 h after exercise compared to maximal values, but the C levels still remained 33.9% higher (*p* < 0.01) than resting values. Similarly, under H5000, C concentrations remained 39.5% (*p* < 0.01) higher 1 h after exercise compared to baseline values (Figure 4). No statistically significant changes in C levels were observed in response to resistance exercise in normoxic and H3000 conditions.

**Figure 5:**
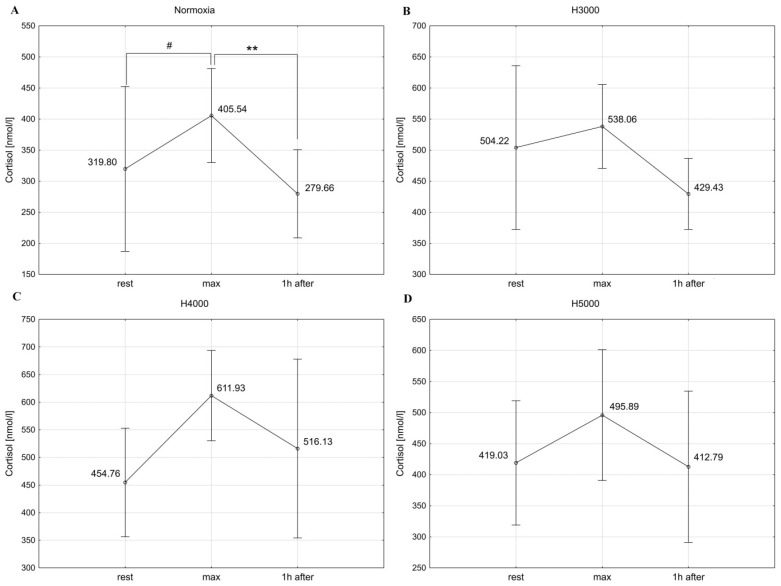
Blood C levels before (rest), immediately after (max), and 1 h after resistance exercise in normoxia (**A**) and hypoxia at simulated altitudes of 3000 m (**B**), 4000 m (**C**), and 5000 m (**D**) in the female group. # *p* < 0.07; ** *p* < 0.01.

In the female group, resistance exercise had a significant effect on C concentrations under normoxia (F = 7.11; *p* < 0.01). Specifically, C levels increased by 26.8% immediately after exercise (*p* < 0.07), which was followed by a drop 1 h after exercise (*p* < 0.01) compared to maximum values. No statistically significant changes were demonstrated in C concentrations in response to exercise in the hypoxic conditions of H3000, H4000, and H5000 (Figure 5).

In the male group, the level of hypoxia affected the magnitude of change in C levels in response to exercise. The increase in C concentration immediately after exercise was significantly higher (*p* < 0.05) under H4000 than under H3000. In the female group, simulated altitudes did not affect the magnitude of change in C concentrations in response to resistance exercise (Table 1).

### 3.4. The T/C Ratio

**Figure 6:**
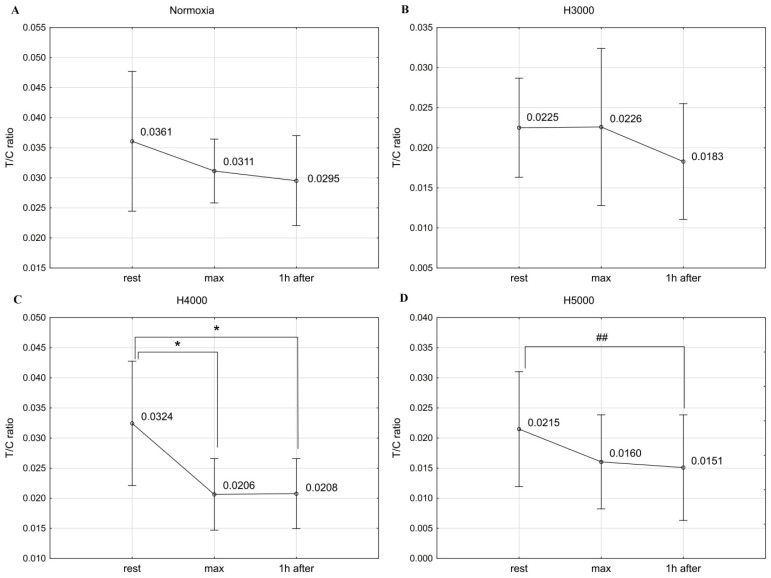
The T/C ratio before (rest), immediately after (max), and 1 h after resistance exercise in normoxia (**A**) and hypoxia at simulated altitudes of 3000 m (**B**), 4000 m (**C**), and 5000 m (**D**) in the male group. ## *p* < 0.06; * *p* < 0.05.

In the male group, resistance exercise led to significant changes in the T/C ratio under H4000 (F = 12.25; *p* < 0.001) and H5000 (F = 4.05; *p* < 0.05). Under H4000, the T/C ratio decreased significantly by 36.4% (*p* < 0.05) immediately after exercise and remained significantly lower (*p* < 0.05) than resting values 1 h after exercise. Under H5000, 1 h after exercise, the T/C ratio showed a drop that was near the accepted level of significance (*p* < 0.06) compared to resting values. Under normoxia and H3000 conditions, no statistically significant changes were observed in the T/C ratio in response to resistance exercise (Figure 6).

**Figure 7:**
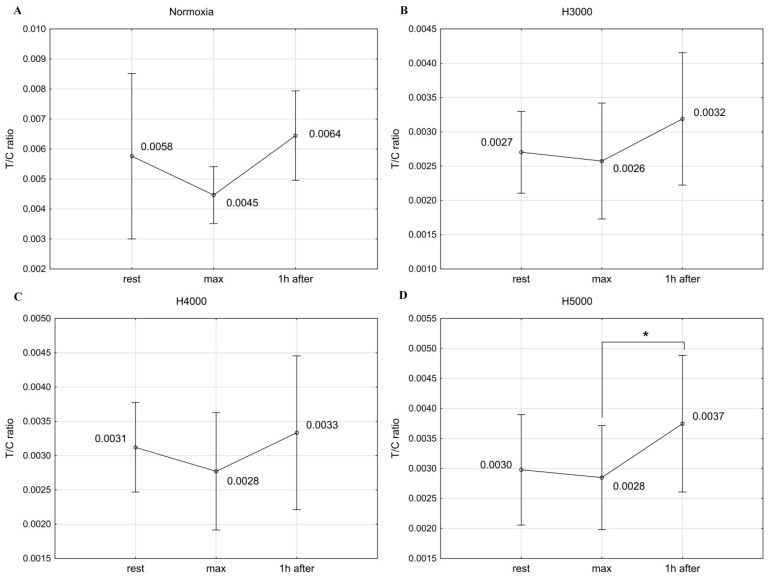
The T/C ratio values before (rest), immediately after (max), and 1 h after resistance exercise in normoxia (**A**) and hypoxia at simulated altitudes of 3000 m (**B**), 4000 m (**C**), and 5000 m (**D**) in the female group. * *p* < 0.05.

In the female group, resistance exercise significantly affected changes in the T/C ratio values under H5000 conditions (F = 4.82; *p* < 0.05). One hour after exercise, the T/C ratio rose significantly (*p* < 0.05) by 32.1% compared to the values recorded immediately after exercise. No statistically significant changes were observed in the T/C ratio in response to exercise under normoxic, H3000, and H4000 conditions (Figure 7).

In the male group, the severity of hypoxia affected the magnitude of change in the T/C ratio in response to exercise. The drop in the T/C ratio immediately after exercise was significantly greater (*p* < 0.05) under H4000 than under H3000. In the female group, simulated altitudes did not affect the magnitude of post-exercise changes in the T/C ratio (Table 1).

### 3.5. Growth Hormone

**Figure 8:**
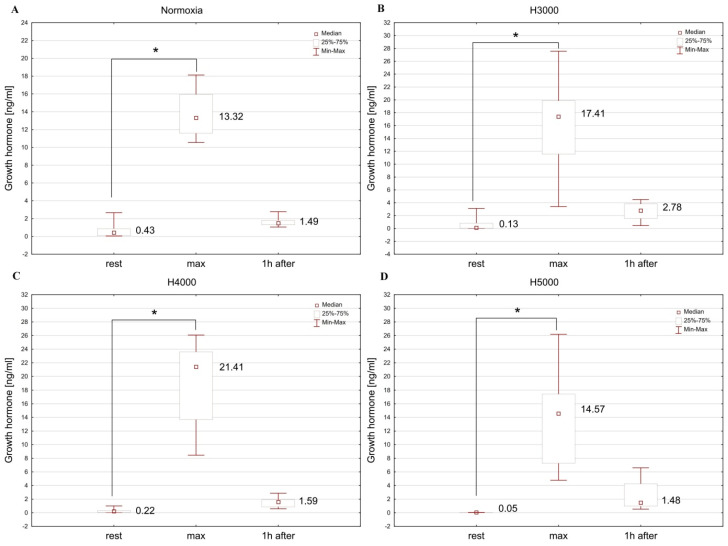
Blood GH levels before (rest), immediately after (max), and 1 h after resistance exercise in normoxia (**A**) and hypoxia at simulated altitudes of 3000 m (**B**), 4000 m (**C**), and 5000 m (**D**) in the male group. * *p* < 0.05.

The male group showed significant differences in GH concentration at consecutive measurement points under normoxia (Chi2 = 14.25; *p* < 0.001), as well as under H3000 (Chi2 = 16.00; *p* < 0.001), H4000 (Chi2 = 16.00; *p* < 0.001), and H5000 (Chi2 = 14.00; *p* < 0.001). Levels of GH increased (*p* < 0.05) immediately after resistance exercise in normoxia and at all simulated altitudes. One hour after exercise, it did not differ significantly from resting levels (Figure 8).

**Figure 9:**
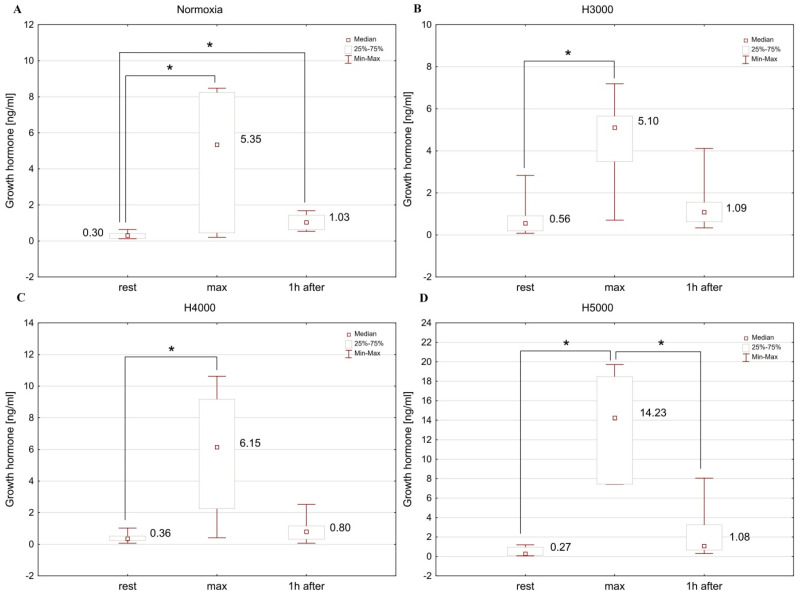
Blood GH levels before (rest), immediately after (max), and 1 h after resistance exercise in normoxia (**A**) and hypoxia at simulated altitudes of 3000 m (**B**), 4000 m (**C**), and 5000 m (**D**) in the female group. * *p* < 0.05.

In the female group, resistance exercise led to significant changes in GH levels in normoxia (Chi2 = 11.14; *p* < 0.01), as well as in H3000 (Chi2 = 7.75; *p* < 0.05), H4000 (Chi2 = 7.00; *p* < 0.05), and H5000 (Chi2 = 11.14; *p* < 0.01). Blood GH concentration increased (*p* < 0.05) immediately after resistance exercise in normoxia and at all simulated altitudes. In hypoxia, GH returned to baseline values 1 h after exercise, but it remained elevated (*p* < 0.05) in normoxia (Figure 9).

In the male group, altitude did not affect the magnitude of post-exercise change in GH levels. In the female group, in contrast, GH concentration was affected by the level of hypoxia: the increase in GH immediately after exercise was significantly greater (*p* < 0.05) under H5000 than under H3000 (Table 1).

### 3.6. Lactate (LA), Creatine Kinase (CK), Lactate Dehydrogenase (LDH), and Uric Acid (UA)

In the male group, the severity of hypoxia significantly affected the magnitude of change in LA (F = 5.19; *p* < 0.01) and CK (F = 4.10; *p* < 0.05). The increase in LA immediately after exercise was significantly smaller under H5000 than under H3000 (*p* < 0.01) and H4000 (*p* < 0.05). In turn, the rise in the CK activity was significantly smaller under H5000 than under H3000 (*p* < 0.05). Altitude had no effect on the magnitude of change in LDH and UA (Table 2).

In the female group, the severity of hypoxia significantly affected the magnitude of change in LA (F = 39.83; *p* < 0.001) and LDH (F = 14.99; *p* < 0.001). The rise in LA levels was significantly greater under H3000 compared to normoxia (*p* < 0.001), H4000 (*p* < 0.01), and H5000 (*p* < 0.001). Furthermore, the increase in LA concentration immediately after exercise under H4000 and H5000 was significantly (*p* < 0.001) greater than in normoxia. In turn, the increase in LDH was significantly greater under H5000 than under normoxia (*p* < 0.001), H3000 (*p* < 0.05), and H4000 (*p* < 0.001). Altitude had no effect on the magnitude of change in CK and UA (Table 2).

## 4. Discussion

The main objective of this study was to analyze the effect of acute normobaric hypoxia of varying severity on the hormonal response to resistance exercise in physically active women and men. Our results indicate that in women, severe hypoxia (H5000) induces a beneficial effect on post-exercise increases in anabolic hormones, particularly GH. In contrast, in men, the use of hypoxia during resistance exercise does not induce more favorable changes in T and GH secretion compared to normoxic conditions. Moreover, in men, severe hypoxia (H5000) leads to a reduction in exercise performance and a weakened metabolic response.

### 4.1. Exercise Capacity and Metabolic Response to Resistance Exercise in Normoxia and Hypoxia

It is believed that one of the key factors influencing the effectiveness of resistance training under hypoxia is the induction of high metabolic stress that triggers muscle adaptations [7,26]. Limited oxygen availability increases reliance on anaerobic metabolism during exercise, enhancing intramuscular metabolic stress and systemic hormonal response [8,27]. For resistance training to be effective, it is crucial that the addition of hypoxia should not negatively impact exercise performance.

Scott et al. [28] demonstrated that the application of a hypoxic stimulus at intensities of 16% and 13% during high-intensity resistance exercise does not alter physical performance during repetitions and sets, nor does it affect the rating of perceived exertion. In contrast, Ramos-Campo et al. [23] found that high-intensity resistance circuit training in high hypoxia (FiO_2_ = 13.0%) causes a significantly greater decline in average and maximal strength in bench press performance across consecutive sets compared to normoxia and lower-intensity hypoxia (FiO_2_ = 16.0%). Our findings revealed that severe hypoxia (H5000, FiO_2_ = 11.2%) limits exercise capacity during high-intensity resistance exercise in men.

The mechanisms causing a decline in exercise performance during repeated bouts of high-intensity resistance exercise under severe hypoxia are not entirely clear. Key factors include hypoxia-induced fatigue at the level of skeletal muscles (peripheral fatigue) as well as the central nervous system (central fatigue) [12]. It is also suggested that a reduced rate of phosphocreatine (PCr) resynthesis during high-intensity resistance exercise with short rest intervals in hypoxic conditions may play a role [12,23].

It is well known that the capacity for exercise performance is progressively elaborated with increased hypoxia. Some studies on resistance training under hypoxic conditions showed an increase in anabolic hormones [14,29]. The hormonal changes that occur during acute exercise in hypoxia seem to be determined by the increase in lactate during exercise, and this effect is more pronounced than regulation by alterations in the hypothalamic-pituitary axis [30].

In our study, the reduction in exercise capacity under severe hypoxia in men was accompanied by a decrease in metabolite accumulation. A trial conducted under H5000 resulted in significantly smaller increases in LA levels and CK activity in response to exercise compared to lower-level hypoxia. Moreover, hypoxic conditions of H3000 (FiO_2_ = 14.4%) and H4000 (FiO_2_ = 12.7%) did not lead to a greater metabolic response compared to normoxia. The existing literature fails to provide clear answers regarding acute LA responses to resistance exercise in hypoxia. Yan et al. [17] reported a rise in LA levels after resistance exercise, but noted no differences between normoxia and hypoxia at 12.6% and 16%. This is consistent with our findings, as well as earlier reports by other authors [15,16]. Contrary to these findings, Kon et al. [13,14] demonstrated that there was a greater rise in LA levels after resistance exercise in severe hypoxia (FiO_2_ = 13%) compared to normoxia, even with low-intensity exercise. Similarly, research by Ramos-Campo et al. [31] found that LA concentrations in severe hypoxia (FiO_2_ = 13%) were higher than in moderate hypoxia (FiO_2_ = 16%) and in normoxia.

Our assumption that hypoxic stress would contribute to increased metabolic stress was confirmed only in the female group. Lactate concentrations increased more after exercise across all levels of hypoxia compared to normoxia. We also observed increased LDH activity after exercise in H5000. Interestingly, women, unlike men, did not experience a reduction in exercise capacity under hypoxic conditions regardless of their severity. This can likely be attributed to the lower external load relative to body mass during exercise.

### 4.2. Changes in Hormone Levels After Resistance Exercise

#### 4.2.1. Testosterone (T)

In the male group, the training protocol contributed to a significant increase in blood T concentrations following resistance exercise under normoxia and H3000. However, similar changes were not observed under H4000 and H5000. Moreover, the severity of hypoxia did not affect the magnitude of the increase in T. These findings align with previous research on hypoxia [13,14,15,17]. For example, Yan et al. [17] used different levels of hypoxia and demonstrated that the T response to resistance exercise did not differ between normoxia and both moderate (FiO_2_ = 16%) and severe (FiO_2_ = 12.6%) hypoxia, suggesting that acute hypoxia may not significantly affect T secretion. Our findings confirm that in the context of the natural release of T following resistance exercise in men, the T response is not directly affected by a short-term hypoxic stimulus. Notably, however, chronic exposure to moderate hypoxia improves T concentrations and the T/C ratio starting from the first week of exposure [32] which is worth keeping in mind in training practice.

Women have resting concentrations of total T that are about 10 times lower than those in men [33], resulting in a correspondingly lower anabolic effect of T. Few studies have reported a transient rise in T following resistance exercise in women [34,35] or no effect on T concentrations during exercise until volitional exhaustion [36]. Our study found that among non-training women who could exercise with a relatively small external load, an increase in T was only triggered by a strong hypoxic stimulus (H4000, H5000). Additionally, under these conditions, T concentrations remained elevated even 1 h after exercise, unlike in men. In the male group, T concentrations returned to baseline or fell below pre-exercise values, which was consistent with trends observed in earlier research [37,38,39,40].

#### 4.2.2. Cortisol (C)

The C response to resistance exercise showed an upward trend in both men and women across all trials. However, in men, statistically significant changes were only observed with strong hypoxic stimuli (H4000 and H5000), and C concentrations remained above baseline values even 1 h after exercise. The literature on the effects of resistance exercise on C concentrations presents mixed findings. Ho et al. [15] reported a decrease in C immediately after exercise in both normoxia and hypoxia (FiO_2_ = 15%). Kurobe et al. [16] revealed that even a strong hypoxic stimulus (FiO_2_ = 12.7%) does not cause significant changes in C concentration. In contrast, a study by Benavente et al. [18] noted an increase in C concentrations at a moderate altitude of 2320 m above sea level. These discrepancies are likely attributable to differences in exercise protocols and loads used. Based on our findings and comparisons of various severities of hypoxia, it can be concluded that in men, a stronger hypoxic stimulus will lead to a more pronounced C response to resistance exercise performed at an intensity of 70% 1RM.

#### 4.2.3. Testosterone-to-Cortisol (T/C) Ratio

A significant rise in C concentrations under severe hypoxia (H4000 and H5000) in men influenced the post-exercise drop in the T/C ratio. The T/C ratio remained lower even 1 h after exercise. However, it is important to note that during this period, the participants remained in hypoxic conditions, which kept blood C concentrations elevated. Combined with the drop in the level of T in blood, this explains the reduction in the T/C ratio values at 1 h post-exercise.

Interestingly, the T/C ratio changes post-exercise exhibited an opposing trend in women compared to men, particularly with severe hypoxia. In men, the T/C ratio remained low 1 h after exercise, whereas in women, the T/C values increased due to a rise in T levels. Future research should investigate whether a sustained favorable T/C ratio in women following resistance exercise under severe hypoxia will translate into enhanced anabolic responses and improved performance in resistance training.

#### 4.2.4. Growth Hormone (GH)

Our study found that GH concentrations rise after resistance exercise in both normoxia and hypoxia for both women and men, with women showing the highest GH secretion under the highest level of hypoxia (H5000). In men, the increase in GH after exercise was similar across all conditions. Previous studies show that resistance training in hypoxia also elevates GH levels, and this increase is comparable to [16,17,18] or even greater than the rise observed in normoxia [13,14,16,17]. However, the difference between GH responses in normoxia and hypoxia may become more pronounced not immediately, but approximately 15–30 min after the end of exercise [16,17].

It was suggested, especially in early research, that exercise and hypoxia might stimulate GH secretion through such mechanisms as increased metabolic stress, reflected, for example, in high LA levels [38,41,42]. However, a significant number of more recent studies have not confirmed the link between rises in LA and GH following resistance exercise [16,17,43]. In our study, men exposed to severe hypoxia (H5000) showed a smaller increase in LA compared to those in lower-level hypoxia (H3000 and H4000), which was associated with a somewhat smaller (though not statistically significant) GH secretion. This was not observed in the female group, where the highest GH secretion was noted under H5000 with a simultaneous smaller increase in LA than under H3000 and H4000. These findings suggest that changes in blood LA levels may not be the primary stimulus behind GH secretion during strength exercise in hypoxia among non-training women.

As suggested in a classical study [44], the regulatory influence of hypoxia on GH levels during exercise is primarily due to changes in relative workout intensity rather than a direct effect of hypoxia itself. Previous studies indicate that one of the mediators of GH action may be the increase in IGF-1, which rises during exercise. Resistance training over six weeks under normobaric hypoxic conditions (FiO_2_ = 12.9%, 4000 m) led to an increase in IGF-1 concentrations at rest following the training period. However, IGF-1 showed no significant changes in response to short-term hypoxic exposure at different altitudes, either with or without exercise [45]. GH release is reduced by alpha-adrenergic blockade and potentiated by beta-adrenergic blockade, demonstrating a possible role of catecholamines in regulating GH secretion [46,47]. In general, adrenergic activity at high altitudes shows an immediate increase, followed by further elevation over the next few days. However, it is worth noting that prolonged exposure to hypoxia leads to greater desensitization and downregulation of beta-adrenergic receptors. Notably, exercise training in hypoxia can, at least partially, prevent this phenomenon [48,49].

It is believed that elevated GH secretion during hypoxia may be indirectly responsible for the ergogenic effects of hypoxia on muscle hypertrophy and strength [50]. Our findings suggest that for women, severe hypoxic conditions (H5000) could help boost muscle mass and strength. However, it is important to remember that individual GH responses to the same resistance training protocol can vary considerably, even within a homogenous population [19,43]. Considerable variations in GH responses to resistance exercise are attributed to factors that include differences in the rate of response of the growth hormone–insulin-like growth factor-1 (GH–IGF-1) axis to the exercise stimulus, which affects differences in the timing of peak plasma GH levels in different individuals [51].

Significant variability in physiological, hormonal, and metabolic responses to both resistance exercise and hypoxia has been repeatedly demonstrated [7,26,28,43], even when the hypoxic stimulus is individualized and SpO_2_ is controlled [22]. In our study, the greatest inter-individual variability was observed in the GH response. In reaction to the hypoxic stimulus, we recorded post-exercise changes in GH concentration ranging from 3.4 ng/mL to 27.4 ng/mL in men and from −0.43 ng/mL to 19.6 ng/mL in women. It has been suggested that variability in blood metabolite and hormone concentrations in response to resistance exercise under different conditions may result from factors beyond hypoxia exposure itself, such as genetic factors or overall physical fitness level [22].

### 4.3. Practical Applications

The response to hypoxia and resistance exercise differs between women and men [19,20,21]. Women possess several physiological traits that significantly influence their response to exercise in hypoxia. The most well-recognized differences include variations in ventilatory control at rest and during exercise [52,53]. This research contributes to the growing body of evidence supporting the distinct anabolic hormone response to hypoxia in women versus men. The higher levels of anabolic hormones observed in women suggest better protection against hypoxia-induced muscle wasting, potentially allowing them to maintain better performance at high altitudes. High testosterone levels are associated with increased hemoglobin levels and, consequently, greater hypoxemia during sleep, which may suggest improved recovery after exercise [54]. Among other factors, these physiological differences can positively impact the effectiveness of the training process, but future studies should further explore the long-term implications of such interventions. Furthermore, these findings should be considered when designing resistance training programs in hypoxic conditions, as both inter-set rest intervals and environmental hypoxia can elicit different hormonal and, consequently, metabolic responses during training sessions.

Attempts have been made in the literature to develop guidelines for resistance training under hypoxia [12,23,26,50,55]; however, these recommendations have been primarily based on studies involving men. Practical applications specifically tailored for women have not yet been established. Although our results require confirmation in further studies involving larger sample sizes, based on our current findings, we can propose the following conclusions for sporting practice:

(1) For women who do not engage in strength-based sports, achieving the high mechanical loads required to cause hypertrophic adaptations is more challenging compared to men, who naturally have greater strength and muscle mass [56]. In women, training under hypoxic conditions may compensate for the relatively lower mechanical loads compared to men, causing greater metabolic stress, while exercise capacity remains preserved.
(2) Severe hypoxia (H5000) appears particularly beneficial for women, as it triggered the greatest rise in anabolic hormones. The increased hormonal response to exercise in hypoxia could potentially enhance the effectiveness of resistance training and strength adaptations in women. Additionally, the use of severe hypoxia might be advantageous for individuals who should avoid high mechanical loads, such as the elderly or athletes returning to regular training after injury.
(3) In men, the application of severe hypoxia during high-intensity resistance exercise may negatively affect exercise performance, leading to a reduction in exercise capacity and a decrease in the total training volume. Consequently, this could limit the development of muscle hypertrophy and strength. Therefore, in practice, resistance training programs should be carefully designed to ensure that hypoxia serves as an additional adaptive stimulus without compromising the metabolic and hormonal responses to exercise. As our study demonstrated, the use of moderate hypoxia (FiO_2_ = 14.4%, H3000) allows for maintaining high exercise performance without negatively affecting metabolic and hormonal reactions.

### 4.4. Limitations

The sample size used in our study allowed us to achieve sufficient statistical power to detect medium and large effect sizes. However, the number of participants was relatively small (8M and 8F), and therefore, caution should be exercised when generalizing the results, particularly considering the inter-individual variability in responses to hypoxia and resistance exercise [26]. Additionally, the limited group size, coupled with differences in the functioning of the hypothalamic–pituitary–gonadal (HPG) axis [57] and the GH–IGF-1 axis [58,59], also provided an argument against statistically comparing the hormonal and metabolic responses between men and women.

A further limitation was the lack of control over the menstrual cycle in the female participants. We relied solely on their declarations that they menstruate regularly and do not use hormonal contraceptives. Previous studies have shown that T levels remain relatively constant in regularly menstruating women who do not use hormonal contraceptives [60,61,62]. Additionally, the menstrual cycle phase does not affect C levels in women who do not take hormonal contraceptives [61,63,64]. GH secretion may be somewhat dependent on resting estrogen concentrations [61,65]. However, a study by Nakamura et al. [61] found no differences in GH release after resistance exercise during the follicular and luteal phases. Given the literature we analyzed and the lack of studies on hormonal responses to resistance exercise in hypoxic conditions in women, we decided to present the data we obtained.

## 5. Conclusions

In men, moderate hypoxia (H3000) is well tolerated and does not impair exercise capacity in terms of muscular strength. However, moderate hypoxia does not enhance post-exercise changes in T and GH levels compared to normoxia. The use of severe hypoxia (H5000) during high-intensity resistance exercise appears unfavorable in men due to reduced metabolic response and diminished exercise capacity, along with a failure to induce more favorable changes in anabolic hormone secretion than in normoxic conditions.

In women, resistance exercise under normobaric hypoxia results in greater metabolic stress compared to normoxia, without negatively affecting exercise capacity. Severe hypoxia (H5000) elicits a pronounced anabolic hormonal response, particularly in GH levels.

## Figures and Tables

**Table 1:** Magnitude of change in T (delta T), C (delta C), the T/C ratio (delta T/C), and GH (delta GH) in response to resistance exercise in normoxia and hypoxia in the male group (M) and in the female group (F).

		Normoxia	H3000	H4000	H5000
Delta T (nmol/L)	M	1.90 ± 1.05	1.55 ± 0.95	1.53 ±3.61	1.15 ± 1.67
	F	0.17 ± 0.21	−0.01 ± 0.11	0.24 ± 0.25 **	0.18 ± 0.16 *
Delta C (nmol/L)	M	152.2 ± 186.7	118.2 ± 169.7	328.3 ± 121.0 *	201.2 ± 107.6
	F	85.7 ± 121.5	33.8 ± 194.5	157.2 ± 153.5	76.9 ± 156.0
Delta T/C	M	−0.0049 ± 0.0095	0.0001 ± 0.0073	−0.0118 ± 0.0066 *	−0.0054 ± 0.0075
	F	−0.0013 ± 0.0028	−0.0001 ± 0.0009	−0.0003 ± 0.0005	−0.0001 ± 0.0009
Delta GH (ng/mL)	M	13.11 ± 3.22	15.44 ± 7.10	18.71 ± 6.24	13.48 ± 7.17
	F	4.64 ± 3.39	3.77 ± 2.15	5.35 ± 4.13	13.33 ± 4.86 *

* *p* < 0.05, ** *p* < 0.01—statistically significant differences compared to H3000.

**Table 2:** The magnitude of change in LA levels (delta LA), the activity of CK (delta CK) and LDH (delta LDH), and the levels of UA (delta UA) in blood in response to resistance exercise in normoxia and hypoxia in the male group (M) and in the female group (F).

		Normoxia	H3000	H4000	H5000
Delta LA (mmol/L)	M	10.2 ± 1.8	11.6 ± 2.9	10.4 ± 1.9	8.3 ± 1.8 ^##^ ^
	F	3.7 ± 1.3	7.4 ± 2.4 ***	6.0 ± 1.3 ^##^ **	5.4 ± 2.4 ^###^ **
Delta CK (U/L)	M	177.0 ± 154.8	172.8 ± 88.9	109.4 ± 71.4	73.9 ± 39.8 ^#^
	F	22.1 ± 15.3	29.3 ± 20.5	17.4 ± 6.9	15.0 ± 5.1
Delta LDH (U/L)	M	19.9 ± 10.8	27.8 ± 5.3	21.1 ± 15.3	13.1 ± 8.6
	F	7.9 ± 4.5	16.0 ± 6.9	6.4 ± 6.5	22.7 ± 15.8 *** ^#^ ^^^
Delta UA (mg/dL)	M	0.02 ± 0.35	0.04 ± 0.16	0.26 ± 0.63	−0.03 ± 0.23
	F	−0.09 ± 0.13	−0.01 ± 0.12	0.05 ± 0.28	−0.14 ± 0.07

** *p* < 0.01; *** *p* < 0.001—statistically significant differences compared to normoxia; # *p* < 0.05; ## *p* < 0.01; ### *p* < 0.001—statistically significant differences compared to H3000; ^ *p* < 0.05; ^^^ *p* < 0.001—statistically significant differences compared to H4000.

## Data Availability

All relevant data for this study are included in the article or available upon reasonable request to the corresponding author.
